# PReS-FINAL-2073: The usefulness of musculoskeletal ultrasound in monitoring efficacy of intraarticular infliximab therapy in JIA patients

**DOI:** 10.1186/1546-0096-11-S2-P85

**Published:** 2013-12-05

**Authors:** M Vidovic, M Perica, L Lamot, L Tambic Bukovac, M Harjacek

**Affiliations:** 1 Childrens Hospital Srebrnjak, Zagreb, Croatia

## Introduction

In the past few years the use of musculoskeletal ultrasound (MSUS) in pediatric rheumatology has proven to be better than clinical examination in detecting synovitis. It is a simple, inexpensive, noninvasive and reproducible method that is ideal for pediatric patient. The progress of therapy options has resulted in quick remission, reversal of inflammation and improvement of life quality; especially with introduction of biologic therapy. To our knowledge this a first report in assessing efficacy of IA infliximab in children with juvenile idiopathic arthritis (JIA) by using MSUS.

## Objectives

To assess the role of MSUS in follow up of patients with JIA treated with intaarticular (IA) infliximab injections.

## Methods

IA infliximab was administered in 6 joints in four patients with JIA. All patients were diagnosed according to ILAR classification criteria and received first and second line therapy (NSAID, DMARD, corticosteroids), with IA corticosteroids on several occasions. None of them fulfilled criteria for biologic therapy but were resistant to DMARD's. The disease was monitored by monthly clinical assessment (JADAS, signs of swelling, pain assessed by patient/parent (VAS), tenderness at palpation and limitation of motion), laboratory tests (ESR, CRP) and MSUS. The Omeract semiquantitative grades (0-3 grades) for both B-mode and Power-Doppler (PD) evaluation of each joint were added and the sum was defined as the Echographic Score (ES). Before IA infliximab (50 mg per joint) was administered, all patients relapsed according to all the assessed factors.

## Results

At the point of IA injections all 6 joints showed grade 3 synovitis in B mode and increased PD signal (3/3). There was also an increase in JADAS, VAS and increased ESR and/or CRP. One month after IA infliximab all patients presented with clinical and gradual US improvement. At the follow-up visit after three months, all of them had grade 1 synovitis without effusion in B mode, PD signal decreased to 0-1/3, and consequently, ES normalized in all joints. There was also clear clinical improvement in JADAS score, local clinical status of injected joints or in ESR and CRP.

## Conclusion

It appears that IA infliximab is very powerful and efficacious medication in selected children with therapy-resistant, isolated mono/oligoarticular JIA. Furthermore, MSUS appears be a valid, sensitive-to-change and feasible method for evaluating joint inflammation, and possible outcome measure in children with JIA treated with IA infliximab.

## Disclosure of interest

None declared.

